# Comparative Efficacy of Different Prehabilitation Strategies in Colorectal Surgery Patients: A Network Meta-Analysis

**DOI:** 10.1016/j.arrct.2025.100523

**Published:** 2025-09-09

**Authors:** Jing-Yi Gao, Jie-Rong Ge, Jing-Yan Cai, Li-Hua Wang

**Affiliations:** a Nantong First People’s Hospital, Operating room, Nantong, Jiangsu Province.; b School of Nursing and Rehabilitation, Nantong University, Nantong, Jiangsu Province.; c Department of Nursing, Nantong First People’s Hospital, Nantong, Jiangsu Province, People's Republic of China.

**Keywords:** Colorectal surgery, Functional capacity, Length of hospital stay, Network meta-analysis, Prehabilitation, Postoperative complications, Readmission, Rehabilitation

## Abstract

**Objectives:**

To evaluate the effect of various prehabilitation strategies on postoperative functional capacity, complication rates, length of hospital stay, and readmission rates in colorectal surgery patients through a network meta-analysis.

**Date Sources:**

A systematic search was performed across PubMed, Embase, Cochrane Library, Web of Science, and Scopus from inception to April 2025.

**Study Selection:**

Randomized controlled trials comparing different prehabilitation strategies (multimodal prehabilitation, exercise-only, nutrition-only, and postoperative rehabilitation) with standard care or against each other were included**.**

**Data Extraction:**

Two investigators independently screened titles/abstracts and assessed full texts for eligibility, with discrepancies resolved through consensus or consultation with a third reviewer. The extracted data included the first author’s name, publication year, country, design, sample size, participants’ age, sex, pathology, surgical procedure, intervention type, and reported outcomes.

**Data Synthesis:**

Fifteen studies involving 1290 participants were included. Compared with standard care, exercise intervention (mean difference, 62.85m; 95% CI, 9.44-116.12m) and multimodal prehabilitation (mean difference, 29.36m; 95% CI, 1.98-67.01m) significantly improved 6-minute walk test performance. Multimodal prehabilitation demonstrated superior efficacy in reducing postoperative complications (odds ratio, 0.65; 95% CI, 0.40-0.96), whereas other intervention methods showed no significant differences compared with standard care. The results of the network meta-analysis indicated that the best probability of improving the 6-minute walk test results was exercise intervention > multimodal prerehabilitation > nutritional intervention > postoperative rehabilitation > standard care. Heterogeneity across studies was low (I²<30%), with minimal risk of publication bias.

**Conclusions:**

Multimodal prehabilitation emerges as the optimal preoperative strategy for colorectal surgery patients, significantly reducing complication risks and enhancing functional capacity. Exercise-only interventions also demonstrate clinically meaningful benefits in functional improvement. Tailored prehabilitation protocols should be implemented based on patient-specific needs and resource availability.

Colorectal cancer remains a leading cause of global cancer-related morbidity and mortality, with surgical resection constituting the primary curative approach.^1^ Despite advancements in surgical techniques and perioperative care, postoperative complication rates persist at approximately 35%, significantly impeding recovery trajectories and escalating health care resource utilization.^2^ Although Enhanced Recovery After Surgery (ERAS) Society protocols have markedly improved postoperative outcomes, perioperative complications continue to pose substantial clinical challenges.^3^

Prehabilitation, an ERAS Society extension, has emerged as a strategy to augment physiological reserve and optimize surgical stress resilience through preoperative interventions.^4^ By enhancing preoperative functional status, prehabilitation may reduce postoperative morbidity, accelerate recovery, and alleviate health care burdens.^5^ Recent years have witnessed a surge in studies exploring diverse prehabilitation modalities for patients who have undergone colorectal surgery, including multimodal programs (integrating exercise, nutrition, and psychological support), exercise-only regimens, and nutrition-focused interventions.^6^

Existing evidence demonstrates the potential benefits of prehabilitation. Gillis et al^7^ reported superior 6 to 8-week postoperative functional capacity in multimodal prehabilitation groups compared with postoperative rehabilitation cohorts. Bousquet-Dion et al^8^ further validated that supervised multimodal prehabilitation accelerates functional recovery. Notably, Molenaar et al’s^9^ multicenter randomized trial established the efficacy of multimodal prehabilitation in reducing postoperative complications.

However, conflicting findings persist. Carli et al^10^ observed no significant reduction in 30-day complications with multimodal prehabilitation versus standard rehabilitation among frail elderly patients with colorectal cancer. Similarly, Onerup et al^11^ found no measurable effect of short-term home-based exercise interventions on complication rates. These discrepancies may stem from heterogeneous study populations, variable intervention protocols, intensity thresholds, and adherence rates.^12^

Crucially, the comparative effectiveness of distinct prehabilitation strategies remains undefined. No studies have directly compared multimodal programs with unimodal interventions, leaving clinicians without evidence-based guidance for protocol selection.^13^ Although conventional meta-analyses have evaluated prehabilitation’s global effects in colorectal surgery.^14^, ^15^, ^16^

Network meta-analysis (NMA), an advanced evidence synthesis methodology, enables simultaneous integration of direct and indirect comparisons to rank multiple interventions—a critical capability for optimizing clinical decision-making.^17^ Unlike conventional pairwise meta-analyses that compare only 2 interventions at a time, NMA simultaneously evaluates all available interventions—even those never directly compared in head-to-head trials—by leveraging indirect evidence across the treatment network. This study, therefore, employs NMA to comprehensively assess the effects of 4 prehabilitation strategies (multimodal, exercise-only, nutrition-only, and postoperative rehabilitation) on functional capacity, complication rates, LOS, and readmission outcomes in colorectal surgery patients. Our findings aimed to establish an evidence hierarchy to guide personalized prehabilitation implementation.

## Methods

This study was conducted in accordance with the Preferred Reporting Items for Systematic Reviews and Meta-Analyses for Network Meta-Analyses guidelines^18^ and the Cochrane Handbook for Systematic Reviews of Interventions.^19^ The protocol was prospectively registered on the PROSPERO international prospective register of systematic reviews (Registration ID: CRD420251044888).

### Literature search strategy

A systematic search was performed across PubMed, Embase, Cochrane Library, Web of Science, and Scopus from inception to April 2025. Search terms included controlled vocabulary (MeSH/Emtree terms) and free-text keywords such as “prehabilitation,” “preoperative rehabilitation,” “preoperative exercise,” “preoperative nutritional support,” “psychological preparation,” “colorectal surgery,” “colorectal cancer,” and “randomized controlled trial,” with adaptations for syntax across databases (see appendix 1 for full strategy). Manual searches of reference lists from included studies supplemented electronic searches to ensure comprehensiveness.

### Eligibility criteria

#### Inclusion criteria

Articles were considered eligible if they met the predefined criteria in terms of participants, interventions, comparators, outcomes, and study design. Inclusion criteria were as follows: (1) population: adults aged (≥18y) scheduled for elective colorectal surgery, irrespective of pathology; (2) interventions: prehabilitation strategies (multimodal [combined exercise, nutrition, and psychological support], exercise-only, and nutrition-only); (3) comparators: alternative prehabilitation strategies, postoperative rehabilitation (rehabilitation interventions initiated exclusively in the postoperative period), or standard care (ERAS Society recommended treament); (4) outcomes: at least 1 primary outcome reported—postoperative functional capacity (eg, 6-minute walk test [6MWT] distance), complication rates (Clavien-Dindo classification ≥ grade II), total hospital length of stay (LOS), or 30-day readmission rates; and (5) study design: randomized controlled trials (RCTs).

#### Exclusion criteria

Exclusion criteria were as follows: (1) nonrandomized designs; (2) duplicate publications or secondary analyses; (3) conference abstracts, reviews, or case reports; (4) interventions lacking explicit prehabilitation components; (5) studies failing to report primary outcomes; and (6) unavailable full texts.

### Exclusion study selection and data extraction

Two investigators (J-Y.G. and J-R.G.) independently screened titles/abstracts and assessed full texts for eligibility, with discrepancies resolved through consensus or consultation with a third reviewer (J-Y.C.). Data were extracted using a standardized template capturing (1) study characteristics: author, publication year, country, design, and sample size; (2) participant details: age, sex, pathology, and surgical procedure; (3) intervention specifics: type, components, intensity, duration, and adherence; (4) comparator details; (5) outcome metrics: 6MWT distance (quantified as change from preoperative baseline to postoperative assessment), complication rates, LOS, and readmission rates; and (6) additional information: attrition rates and funding sources. Corresponding authors were contacted for missing data.

### ROB assessment

Methodological quality was evaluated using the revised Cochrane Risk of Bias (ROB) Tool 2.0,^20^^, a^ assessing 5 domains: randomization process, deviations from intended interventions, missing outcome data, outcome measurement, and selective reporting. Two reviewers independently classified each domain as “low risk,” “some concerns,” or “high risk,” with overall judgments synthesized into domain-specific risk plots.

### Data synthesis and statistical analysis

#### Network geometry

Intervention nodes (multimodal prehabilitation, exercise-only, nutrition-only, postoperative rehabilitation, standard care) were connected by edges representing direct comparisons, weighted by study count and node size by sample size.

#### Effect size calculation

Continuous outcomes (6MWT, LOS) were analyzed using mean differences (MDs) with 95% CIs. Dichotomous outcomes (complications and readmissions) were assessed using odds ratios (ORs). Medians and interquartile ranges were converted to means and standard deviations using Wan et al’s ^21^ method.

#### Heterogeneity assessment

We tested whether the study results were similar enough to combine using standard statistical measures. Studies with similar results (I²<25%) were considered to have low variation, whereas those with I²>50% were considered to have high variation. Subgroup analyses explored sources of differences between studies.

#### NMA

We used appropriate statistical models based on how similar the study results were. When studies showed similar results (I²<50%), we used simpler models; when results varied more, we used more flexible models. Multiarm studies were adjusted using established statistical methods.

#### Consistency evaluation

We checked whether direct and indirect evidence gave similar results. When studies directly compared 2 treatments, this provided “direct evidence.” When we could infer comparisons through other studies (for example, if study A compared treatment 1 vs 3, and study B compared treatment 2 vs 3, we could indirectly compare treatments 1 and 2), this provided “indirect evidence.” Consistency was assumed when these 2 types of evidence agreed.

#### Ranking probabilities

We ranked treatments from best to worst using a standardized scoring system called surface under the cumulative ranking curve (SUCRA) scores. SUCRA scores range from 0% to 100%, with higher scores indicating better treatments. A score of 100% means the treatment is most likely to be the best, whereas 0% means it is most likely to be the worst.

#### Publication bias

Comparison-adjusted funnel plots and Egger’s test evaluated small-study effects. The trim-and-fill method (R “netfunnel” package^22^^, b^) adjusted for potential asymmetry.^23^

## Results

### Literature screening process

**Fig 1:**
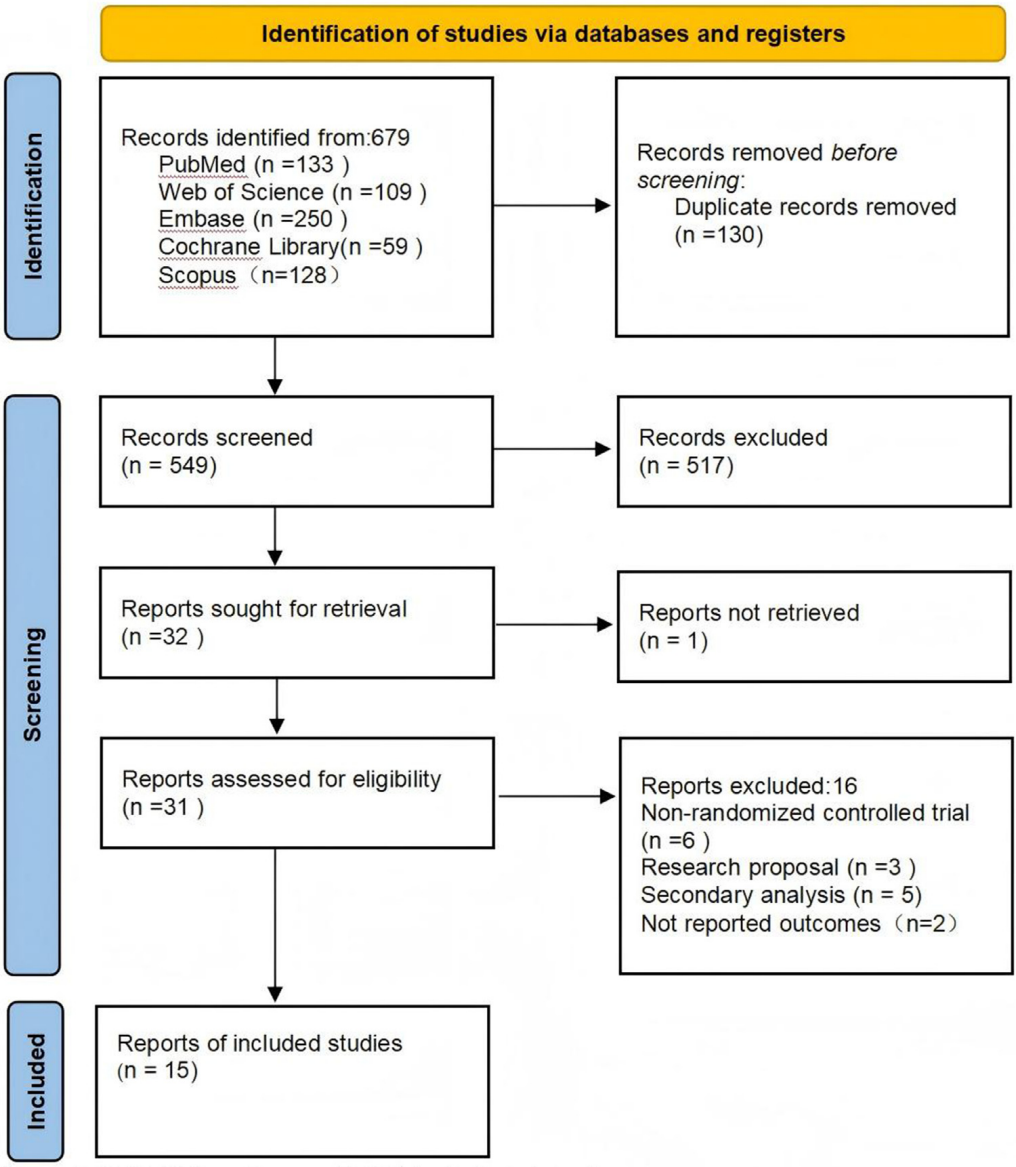
Prisma flow diagram (2020) for included studies.

A total of 551 studies were initially identified, all published in English. After removing duplicates, 446 studies remained. Title and abstract screening yielded 32 articles, among which those failing to meet the inclusion criteria or with unavailable data were excluded. Ultimately, 15 studies were included in the final analysis. The literature selection process and results are presented in figure 1.

### Characteristics of included studies

This NMA included 15^7-10,24-34^ RCTs published between 2014 and 2024, all focusing on prehabilitation interventions for patients who underwent colorectal cancer surgery. Sample sizes ranged from 20 to 251 participants, with a median of 63 participants, totaling 1290 participants across all studies. Interventions included multimodal prehabilitation (10 studies,^7^, ^8^, ^9^, ^10^^,^^25^^,^^26^^,^^28^^,^^32^, ^33^, ^34^ and 916 participants), exercise interventions (4 studies,^24^^,^^29^, ^30^, ^31^ 331 participants), and nutritional intervention (1 study,^27^ 43 participants), whereas control groups primarily received standard care (12 studies^9^^,^^24^, ^25^, ^26^, ^27^, ^28^, ^29^, ^30^, ^31^, ^32^, ^33^, ^34^, 1040 participants) or postoperative rehabilitation (3 studies,^7^^,^^8^^,^^10^ 250 participants). All study populations consisted of patients undergoing colorectal surgery, with 1 study conducted during the coronavirus disease of 2019 pandemic. Five studies evaluated home-based prehabilitation programs, and 2 studies focused on the long-term effects of short-term prehabilitation protocols.

### ROB assessment

**Fig 2:**
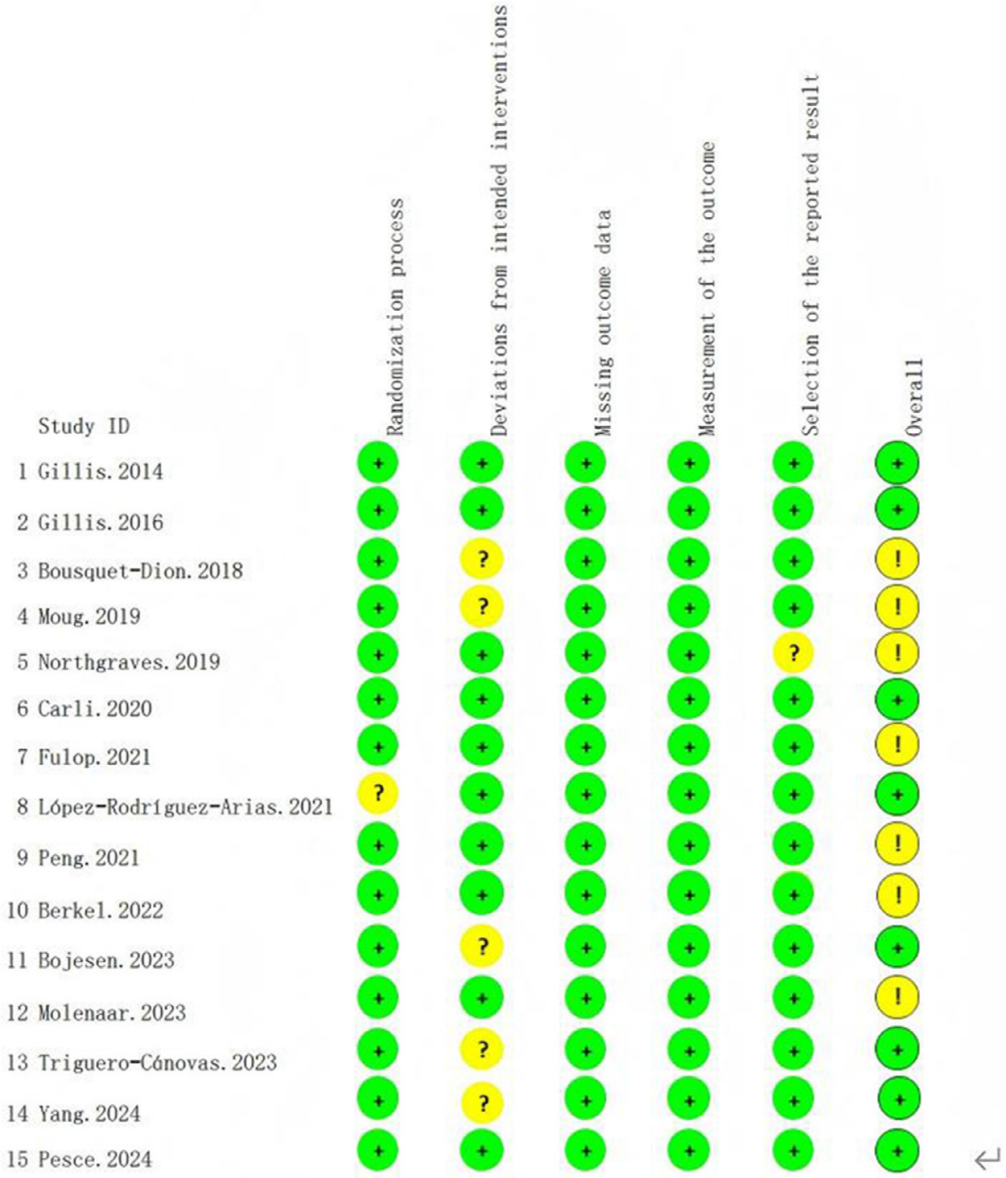
The ROB summary for studies included in the meta-analysis.

Using the Cochrane ROB assessment tool, most included studies demonstrated a low overall ROB (fig 2). Regarding the randomization process, 14 studies^7^, ^8^, ^9^, ^10^^,^^24^, ^25^, ^26^, ^27^^,^ 29, 30, 31, 32, 33, 34 were assessed as low risk, with only 1 study^28^ having an uncertain risk. For deviations from intended interventions, 10 studies^7^^,^^9^^,^^10^^,^^24^^,^^26^, ^27^, ^28^^,^^30^, ^31^, ^32^ presented low risk, whereas 5 studies^8^^,^^25^^,^^29^^,^^33^^,^^34^ had uncertain risk. All studies were rated as low risk for missing outcome data, indicating excellent data integrity across included research. Similarly, all studies were assessed as low risk regarding outcome measurement, suggesting appropriate outcome assessment methods were used. For selective reporting of results, 14 studies^7^, ^8^, ^9^, ^10^^,^^24^, ^25^, ^26^, ^27^, ^28^, ^29^^,^^31^, ^32^, ^33^, ^34^ were evaluated as low risk, with 1 study^30^ showing uncertain risk. Overall, 8 studies^7^^,^^10^^,^^25^^,^^27^^,^^28^^,^^32^, ^33^, ^34^ were classified as low ROB, 7 studies^8^^,^^9^^,^^24^^,^^26^^,^^29^, ^30^, ^31^ had some concerns, and no studies were classified as high ROB, demonstrating good overall quality of the included evidence.

### 6MWT

**Fig 3:**
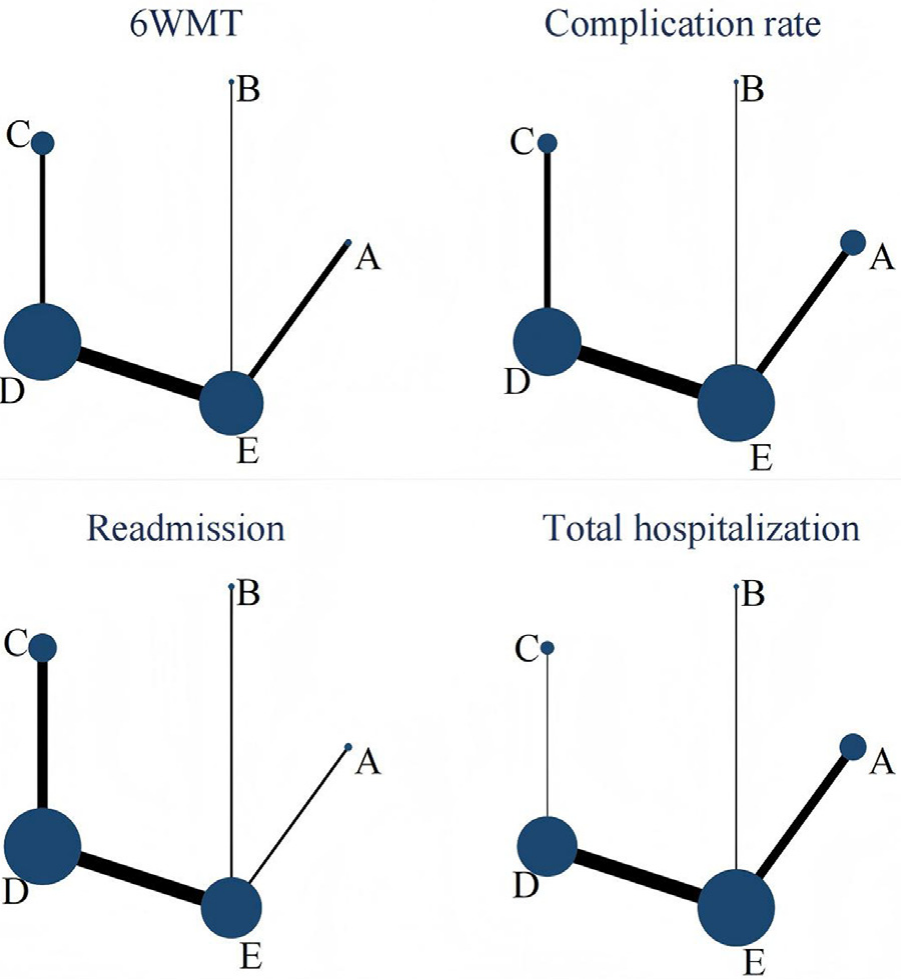
Network plot. A, exercise intervention; B, nutritional intervention; C, postoperative rehabilitation; D, multimodal prehabilitation; E, standard care.

The NMA for the 6MWT showed moderate heterogeneity (I²=27.6%, *P*=.136). As no closed loops were formed, consistency analysis could not be performed; however, visual comparison of consistency and inconsistency models revealed no notable differences, justifying the use of a consistency model for analysis (fig 3).

Results showed that compared with standard care, exercise interventions significantly improved postoperative 6MWT distances (MD, 62.85m; 95% CI, 9.44-116.12m) (table 1), as did multimodal prehabilitation (MD, 29.36m; 95% CI, 1.98-67.01m). According to SUCRA score rankings, exercise interventions ranked first (SUCRA score=80%), followed by multimodal prehabilitation (SUCRA score=55%), nutritional intervention (SUCRA score=27%), postoperative rehabilitation (SUCRA score=23%), and standard care (SUCRA score=1%). Notably, these point estimates exceed the clinically meaningful threshold of ≥20 m for functional recovery after colorectal surgery.^32^ This indicates that exercise interventions and multimodal prehabilitation are most effective in improving patients’ functional capacity.

**Table 1:** NMA results of 6WMT.

Prehabilitation Strategy	Exercise Intervention	Nutritional Intervention	Postoperative Rehabilitation	Multimodal Prehabilitation	Standard Care
Exercise intervention		−43.92 (−129.59 to 40.38)	−59.05 (−127.46 to 16.93)	−32.66 (−90.92 to 33.59)	−62.85 (−116.12 to -9.44)
Nutritional intervention	43.92 (−40.38 to 129.59)		−15.64 (−92.86 to 70.83)	10.35 (−52.77 to 88.83)	−18.68 (−82.10 to 45.29)
Postoperative rehabilitation	59.05 (−16.93 to 127.46)	15.64 (−70.83 to 92.86)		26.19 (−10.71 to 65.30)	−3.27 (−58.14 to 42.05)
Multimodal prehabilitation	32.66 (−33.59 to 90.92)	−10.35 (−88.83 to 52.77)	−26.19 (−65.30 to 10.71)		−29.36 (−67.01 to −1.98)
Standard care	62.85 (9.44-116.12)*	18.68 (−45.29 to 82.10)	3.27 (−42.05 to 58.14)	29.36 (1.98-67.01)*	_

Interventions are compared pairwise. For example, exercise vs standard care shows a mean improvement of 62.85 m (95% CI, 9.44-116.12) favoring exercise.

⁎ , Statistically significant.

**Fig 4:**
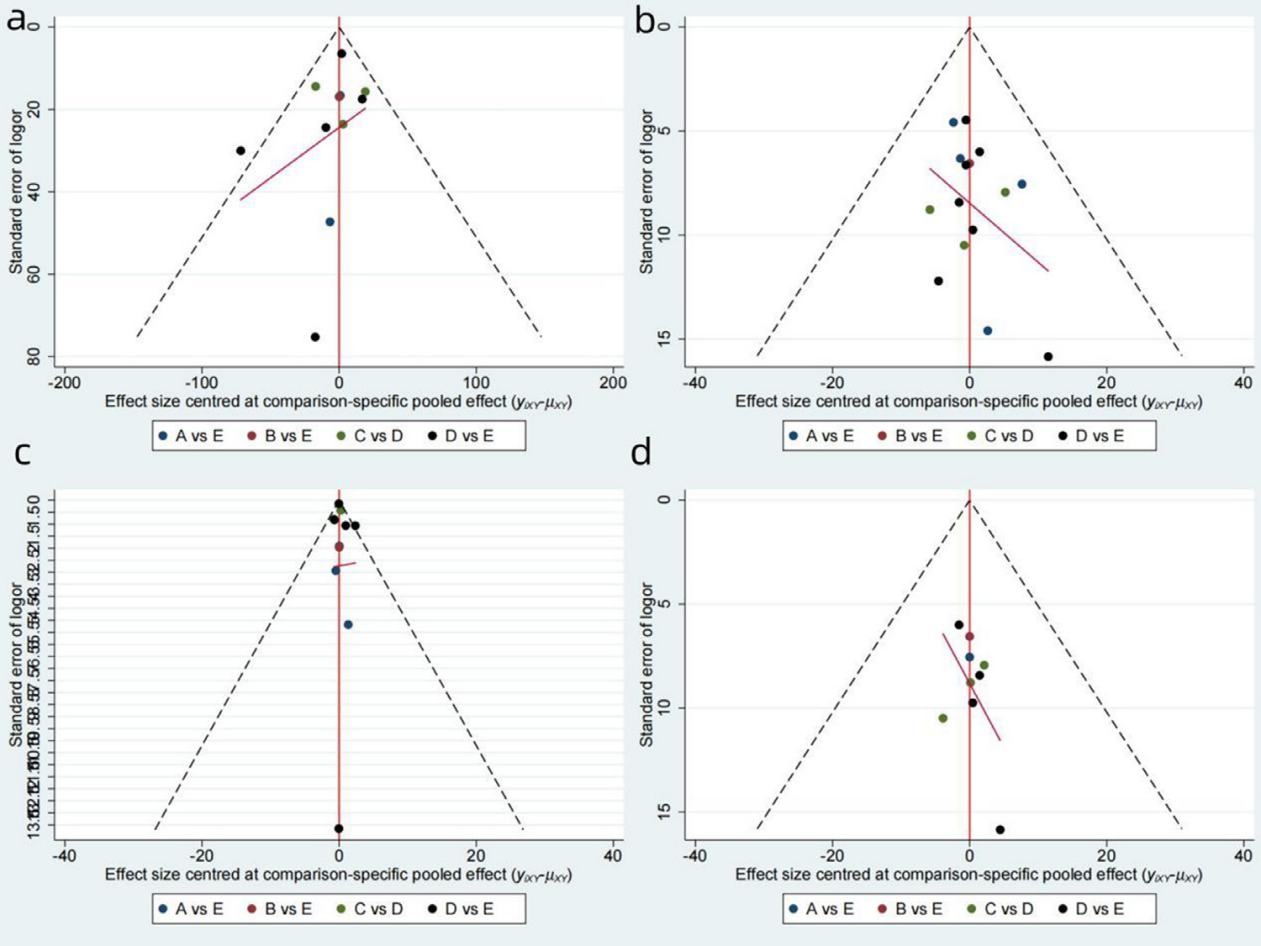
(A) Funnel plot of 6MWT. (B) Funnel plot of complication rate. (C) Funnel plot of hospitalization days. (D) Funnel plot of readmission rate. A, exercise intervention; B, nutritional intervention; C, postoperative rehabilitation; D, multimodal prehabilitation; E, standard care.

Funnel plot analysis showed no apparent asymmetry (Egger test, *P*=.843) (fig 4A), suggesting the absence of publication bias. Comparing pre-adjustment and postadjustment analysis results, conclusions remained stable, further supporting the reliability of the findings.

### Complication rate

The NMA for complication rates revealed low heterogeneity (I²=20.5%, *P*=.192). Without closed loops, consistency analysis could not be conducted; however, visual comparison of consistency and inconsistency models showed no notable differences, supporting the use of a consistency model (fig 3).

Results demonstrated that multimodal prehabilitation significantly reduced complication risk compared with standard care (OR, 0.65; 95% CI, 0.40-0.96) (table 2). Other interventions showed no significant differences compared with standard care: exercise interventions (OR, 0.68; 95% CI, 0.41-1.39), nutritional intervention (OR, 0.97; 95% CI, 0.27-5.48), and postoperative rehabilitation (OR, 0.64; 95% CI, 0.32-1.49). SUCRA scores indicated standard care ranked worst (SUCRA score=0%), followed by nutritional intervention (SUCRA score=15%), postoperative rehabilitation (SUCRA score=28%), multimodal prehabilitation (SUCRA score=28%), and exercise interventions (SUCRA score=29%). This study suggests multimodal prehabilitation is the most effective strategy for reducing postoperative complications.

**Table 2:** NMA results of complication rate.

Prehabilitation Strategy	Exercise Intervention	Nutritional Intervention	Postoperative Rehabilitation	Multimodal Prehabilitation	Standard Care
Exercise intervention		1.51(0.33-8.71)	1.00 (0.34-2.28)	0.93 (0.39-1.73)	1.46 (0.72-2.45)
Nutritional intervention	0.66 (0.11-3.06)		0.67 (0.11-2.90)	0.65 (0.11-2.27)	1.03 (0.18-3.64)
Postoperative rehabilitation	1.00 (0.44-2.96)	1.50 (0.34-9.36)		0.97 (0.50-1.65)	1.57 (0.67-3.12)
Multimodal prehabilitation	1.08 (0.58-2.58)	1.55 (0.44-9.00)	1.03 (0.60-2.01)		1.54 (1.04-2.52)*
Standard care	0.68(0.41-1.39)	0.97 (0.27-5.48)	0.64 (0.32-1.49)	0.65(0.40-0.96)*	

Interventions are compared pairwise. For example, multimodal prehabilitation vs standard care shows an odds ratio of 0.65 (0.40-0.96) favoring standard care.

⁎ , Statistically significant.

Funnel plot analysis for complication rates exhibited symmetrical distribution (Egger test, *P*=.712) (fig 4B), indicating no significant publication bias. Comparing results before and after trim-and-fill adjustments, effect size estimates remained stable, further supporting the reliability of the analysis.

### Total hospitalization days

The NMA for total hospitalization days demonstrated low heterogeneity (I²=18.9%, *P*=.228). Without closed loops, consistency analysis could not be performed; however, visual comparison showed no notable differences between consistency and inconsistency models, justifying the use of a consistency model (fig 3).

Results indicated that none of the prehabilitation interventions showed statistically significant differences in reducing hospital stay compared with standard care: exercise interventions (MD, −0.56d; 95% CI, −3.89 to 2.69d) (table 3), nutritional intervention (MD, −1.05d; 95% CI, −5.13 to 3.53d), postoperative rehabilitation (MD, 0.05d; 95% CI, −2.27 to 2.13d), and multimodal prehabilitation (MD, −0.31d; 95% CI, −1.86 to 0.79d). According to SUCRA scores, standard care ranked worst (SUCRA = 2%), whereas exercise interventions ranked best (SUCRA score=44%), followed by nutritional intervention (SUCRA score=38%), multimodal prehabilitation (SUCRA score=9%), and postoperative rehabilitation (SUCRA score=7%). Despite nonsignificant differences, exercise interventions showed potential advantages in reducing hospital stay.

**Table 3:** NMA results of total hospitalization days.

Prehabilitation Strategy	Exercise Intervention	Nutritional Intervention	Postoperative Rehabilitation	Multimodal Prehabilitation	Standard Care
Exercise intervention		−0.4.(−5.71 to 5.13)	0.56 (−3.34 to 4.51)	0.21 (−3.34 to 3.73)	0.56 (−2.69 to −3.89)
Nutritional intervention	0.40 (−5.13 to 5.71)		1.03 (−4.05 to 5.79)	0.69 (−4.15 to 4.94)	1.05 (−3.53 to 5.13)
Postoperative rehabilitation	−0.56 (−4.51 to 3.34)	−1.03 (−5.79 to 4.05)		−0.36 (−2.27 to 1.30)	−0.05 (−2.13 to 2.27)
Multimodal prehabilitation	−0.21 (−3.73 to 3.34)	−0.69 (−4.94 to 4.15)	0.36 (−1.30 to 2.27)		0.31 (−0.79 to −1.86)
Standard care	−0.56 (−3.89 to 2.69)	−1.05 (−5.13 to 3.53)	0.05 (−2.27 to 2.13)	−0.31 (−1.86 to 0.79)	

Interventions are compared pairwise. Results indicated that none of the prehabilitation interventions showed statistically significant differences in reducing hospital stay compared with standard care.

Funnel plot analysis showed no apparent asymmetry (Egger test, *P*=.784) (fig 4C), suggesting the absence of publication bias. Sensitivity analysis, conducted by excluding studies 1 by 1 and reanalyzing, yielded stable results, further supporting the reliability of the findings.

### Readmission rate

The NMA for readmission rates showed low heterogeneity (I²=24.7%, *P*=.195). Without closed loops, consistency analysis could not be performed; however, visual comparison revealed no notable differences between consistency and inconsistency models, supporting the use of a consistency model (fig 3).

Results indicated that no intervention demonstrated statistically significant differences in reducing readmission rates compared with standard care: exercise interventions (OR, 0.79; 95% CI, 0.12-5.11) (table 4), nutritional intervention (OR, 2.89; 95% CI, 0.41-34.02), postoperative rehabilitation (OR, 0.86; 95% CI, 0.16-3.95), and multimodal prehabilitation (OR, 0.82; 95% CI, 0.27-2.44). According to SUCRA scores, nutritional intervention ranked worst (SUCRA score=73%), followed by standard care (SUCRA score=33%), postoperative rehabilitation (SUCRA score=20%), exercise interventions (SUCRA score=10%), and multimodal prehabilitation ranked best (SUCRA score=3%). Despite nonsignificant differences, multimodal prehabilitation showed some advantage in reducing readmission rates.

**Table 4:** Network meta-analysis results of readmission rate.

Prehabilitation Strategy	Exercise Intervention	Nutritional Intervention	Postoperative Rehabilitation	Multimodal Prehabilitation	Standard Care
Exercise intervention		4.28 (0.23-76.41)	1.00 (0.08-12.02)	1.00 (0.11-9.82)	1.27 (0.20-.53)
Nutritional intervention	0.23 (0.01-4.31)		0.27 (0.02-3.45)	0.26 (0.02-2.75)	0.35 (0.03-2.42)
Postoperative rehabilitation	1.00 (0.08-12.60)	3.76 (0.29-66.33)		0.95 (0.33-2.96)	1.57 (0.25-6.21)
Multimodal prehabilitation	1.00 (0.10-9.34)	3.78 (0.36-56.10)	1.05 (0.34-2.99)		1.21 (0.41-3.76)
Standard care	0.79 (0.12-5.11)	2.89 (0.41-34.02)	0.86 (0.16-3.95)	0.82 (0.27-2.44)	

Interventions are compared pairwise. Results indicated that no intervention demonstrated statistically significant differences in reducing readmission rates compared with standard care.

Funnel plot analysis for readmission rates exhibited good symmetry (Egger test, *P*=.871) (fig 4D), indicating no significant publication bias. Results after small-study adjustments were similar to original findings, further supporting the reliability of the results.

## Discussion

This NMA evaluated the efficacy of different prehabilitation strategies in patients undergoing colorectal cancer surgery, including 15 RCTs with 1290 participants. Our findings revealed that exercise interventions and multimodal prehabilitation significantly outperformed standard care in improving postoperative 6-MWT performance, whereas multimodal prehabilitation was the only intervention that significantly reduced postoperative complication rates. Regarding length of hospital stay and readmission rates, although differences between interventions and standard care did not reach statistical significance, exercise interventions showed potential advantages in reducing hospitalization duration, whereas multimodal prehabilitation demonstrated some benefit in lowering readmission rates.

Our results align with previous meta-analyses, such as the study by Gillis et al,^7^ which also found that multimodal prehabilitation reduced postoperative complication risk and shortened hospital stays. However, through NMA methodology, our study is the first to directly compare the relative effects of different prehabilitation strategies, revealing that exercise interventions may surpass multimodal prehabilitation in improving functional capacity. This finding is consistent with Thomas et al,^35^ who identified exercise training as the core component of prehabilitation. Notably, similar to Minnella et al,^5^ we found that nutritional interventions alone showed limited efficacy but may produce synergistic effects when incorporated into multimodal prehabilitation. Carli et al^4^ further supported this perspective, emphasizing that combining exercise and nutritional interventions may produce additive effects.

Improved functional capacity emerged as one of the most significant findings in our study, consistent with Levett et al_,_^36^ confirming that prehabilitation can markedly enhance patients’ physiological reserve, thereby strengthening their ability to cope with surgical stress. In their recent research, Molenaar et al^9^ also highlighted that enhanced functional capacity may be a key mechanism through which multimodal prehabilitation reduces complication risk. Regarding complication reduction, our results align with Onerup,^11^ indicating that structured prehabilitation interventions effectively reduce specific types of postoperative complications.

### Study limitations

The strengths of this study include its statsus as the first NMA, to the best of our knowledge that directly compares multiple prehabilitation strategies while assessing several key clinical outcomes. Additionally, there is low heterogeneity among the included studies, which enhances the reliability of the results, and all included studies demonstrate a low ROB. Nevertheless, limitations remain: some intervention groups had small sample sizes, particularly with only 1 study for nutritional intervention, which, combined with the inherently modest effect sizes of prehabilitation interventions, contributed to the wide confidence intervals observed in several comparisons (eg, OR for complications, 0.64; 95% CI, 0.32-1.49); detailed information on intervention duration, intensity, and adherence was lacking, limiting dose-response relationship analysis; potential influencing factors such as surgery type, age, and comorbidities were not considered; and most studies had relatively short follow-up periods, preventing long-term effect assessment. These are common methodological issues in prehabilitation research, as highlighted by van Rooijen et al.^37^

Based on our findings, multimodal prehabilitation should be prioritized for patients undergoing colorectal cancer surgery, especially those at high risk for complications; when resources are limited, exercise interventions can serve as effective alternatives for improving functional capacity; and prehabilitation strategy selection should be individualized based on patient-specific needs and available resources. This aligns with the concept of personalized prehabilitation protocols proposed by Scheede-Bergdahl et al.^6^ Multimodal prehabilitation likely represents the optimal choice for comprehensively improving postoperative recovery, particularly in reducing complications and enhancing functional capacity, consistent with the latest guidelines from the ERAS Society.^3^

Our study encompassed all patients undergoing colorectal surgery, with a primary focus on those diagnosed with cancer. This constitutes a notable limitation, as the preoperative trajectory and functional status may differ considerably between oncological patients and other clinical groups, such as individuals with inflammatory bowel disease. Further research should investigate these variations to enhance the understanding of multimodal prehabilitation’s effect across diverse patient cohorts.

Future research should explore individualized prehabilitation protocols for different patient populations; evaluate optimal timing, duration, and intensity of prehabilitation, as Barberan-Garcia et al^38^ emphasized that these factors may significantly affect prehabilitation effectiveness; conduct more studies on nutritional interventions, especially comparing different nutritional supplementation regimens; perform longer-term follow-up studies to assess prehabilitation effects on long-term functional recovery and quality of life; and investigate the effectiveness of remote monitoring and home-based prehabilitation, particularly in resource-limited settings or special circumstances (such as pandemics), which López-Rodríguez-Arias et al^28^ have shown preliminary feasibility in their research.

## Conclusions

This NMA demonstrated that multimodal prehabilitation represents the optimal strategy for preoperative preparation in patients undergoing colorectal cancer surgery, significantly reducing postoperative complication risk while improving functional capacity. Exercise interventions also show significant effectiveness in enhancing postoperative functional capacity. Although differences among intervention strategies regarding hospital LOS and readmission rates did not reach statistical significance, the overall trends support the clinical value of multimodal prehabilitation and exercise interventions. In clinical practice, appropriate prehabilitation strategies should be selected based on patient characteristics and health care environment, with multimodal prehabilitation incorporating exercise, nutrition, and psychological interventions likely providing the most comprehensive benefits. Future research should focus on the individualized customization of prehabilitation protocols and the assessment of their long-term effects.

## Suppliers

a. Risk of Bias Tool 2.0; Cochrane.
b. R; The R Project for Statistical Computing.

## Disclosure

The investigators have no financial or nonfinancial disclosures to make in relation to this project.

## Authorship Contributions/CRediT statements

1. Jing-Yi Gao: Topic concept, literature search, data analysis, and article writing.
2. Jie-Rong Ge: Literature search and data analysis.
3. Jing-Yan Cai: Literature search and data analysis.
4. Li-Hua Wang: Revise and review articles.

## Data statements

All the data are available from the corresponding author up on a reasonable request.

## Appendix 1 Search Strategy for PubMed

#1

"Colorectal Surgery"[Mesh] OR "Colorectal Neoplasms/surgery"[Mesh] OR "Colonic Neoplasms/surgery"[Mesh] OR "Rectal Neoplasms/surgery"[Mesh] OR "Colectomy"[Mesh] OR "Colorectal Surgery"[tiab] OR "Colorectal Resection"[tiab] OR "Colon Resection"[tiab] OR "Rectal Resection"[tiab] OR "Colon Surgery"[tiab] OR "Rectal Surgery"[tiab] OR "Colectomy"[tiab] OR "Colorectal Operation"[tiab] OR "Bowel Surgery"[tiab] OR "Intestinal Surgery"[tiab] OR ((colorectal[tiab] OR colon[tiab] OR rectal[tiab] OR rectum[tiab] OR bowel[tiab] OR intestin*[tiab]) AND (surgery[tiab] OR surgical[tiab] OR operation[tiab] OR resection[tiab] OR operative[tiab]))

#2

"Prehabilitation"[Mesh] OR prehabilitat*[tiab] OR pre-habilitat*[tiab] OR "pre habilitat*"[tiab] OR "preoperative rehabilitation"[tiab] OR "pre-operative rehabilitation"[tiab] OR "preoperative exercise"[tiab] OR "pre-operative exercise"[tiab] OR "preoperative training"[tiab] OR "pre-operative training"[tiab] OR "preoperative conditioning"[tiab] OR "pre surgery preparation"[tiab] OR "presurgical preparation"[tiab] OR "pre-surgical preparation"[tiab] OR "presurgical intervention"[tiab] OR "pre-surgical intervention"[tiab] OR "preoperative intervention"[tiab] OR "pre-operative intervention"[tiab] OR "preoperative program"[tiab] OR "pre-operative program"[tiab] OR "preoperative preparation"[tiab]

#3

("Multimodal"[tiab] OR "Multi-modal"[tiab] OR "Multi modal"[tiab] OR "Trimodal"[tiab] OR "Three-component"[tiab] OR "Multiple component"[tiab] OR "Combined"[tiab] OR "Comprehensive"[tiab] OR "Integrative"[tiab]) AND (#2)

#4

("Exercise"[Mesh] OR "Exercise Therapy"[Mesh] OR "Physical Fitness"[Mesh] OR "Exercise"[tiab] OR "Physical Activity"[tiab] OR "Training"[tiab] OR "Fitness"[tiab] OR "Physical Therapy"[tiab] OR "Physiotherapy"[tiab] OR "Aerobic"[tiab] OR "Resistance Training"[tiab] OR "Strength Training"[tiab] OR "Endurance Training"[tiab] OR "Walking"[tiab] OR "Physical Conditioning"[tiab] OR "Exercise Prescription"[tiab] OR "Exercise Program"[tiab]) AND (#2)

#5

("Nutrition Therapy"[Mesh] OR "Diet Therapy"[Mesh] OR "Nutritional Support"[Mesh] OR "Dietary Supplements"[Mesh] OR "Protein Supplements"[tiab] OR "Nutrition"[tiab] OR "Nutritional"[tiab] OR "Nutritional Support"[tiab] OR "Nutritional Supplementation"[tiab] OR "Dietary Supplement"[tiab] OR "Diet"[tiab] OR "Dietary"[tiab] OR "Protein"[tiab] OR "Proteins"[tiab] OR "Oral Nutritional Supplement"[tiab] OR "ONS"[tiab] OR "Food Supplement"[tiab] OR "Dietary Counseling"[tiab] OR "Nutritional Counseling"[tiab] OR "Diet Therapy"[tiab] OR "Alimentary"[tiab] OR "Feeding"[tiab] OR "Energy Intake"[tiab] OR "Protein Intake"[tiab] OR "Caloric Intake"[tiab] OR "Malnutrition"[tiab] OR "Nutrition Assessment"[tiab] OR "Enteral Nutrition"[tiab]) AND (#2)

#6

#3 OR #4 OR #5 OR #2

#7

"Randomized Controlled Trial"[pt] OR "Controlled Clinical Trial"[pt] OR "Randomized"[tiab] OR "Randomised"[tiab] OR "Randomly"[tiab] OR "Trial"[tiab] OR "Groups"[tiab] OR "Clinical Trials as Topic"[Mesh] OR "Random Allocation"[Mesh] OR "Therapeutic Use"[sh]

#8

#1 AND #6 AND #7

## Appendix B Supplementary materials

Image, application 1
