# Supplementary material for: Comparative Efficacy of Different Prehabilitation Strategies in Colorectal Surgery Patients: A Network Meta-Analysis
Source: Arch Rehabil Res Clin Transl. 2025 Sep 9;7(4):100523. doi: 10.1016/j.arrct.2025.100523 (PMC12750349; doi:10.1016/j.arrct.2025.100523)
Supplement: Supplementary file 1 [file mmc1.docx]

#1

"Colorectal Surgery"[Mesh] OR "Colorectal Neoplasms/surgery"[Mesh] OR "Colonic Neoplasms/surgery"[Mesh] OR "Rectal Neoplasms/surgery"[Mesh] OR "Colectomy"[Mesh] OR "Colorectal Surgery"[tiab] OR "Colorectal Resection"[tiab] OR "Colon Resection"[tiab] OR "Rectal Resection"[tiab] OR "Colon Surgery"[tiab] OR "Rectal Surgery"[tiab] OR "Colectomy"[tiab] OR "Colorectal Operation"[tiab] OR "Bowel Surgery"[tiab] OR "Intestinal Surgery"[tiab] OR ((colorectal[tiab] OR colon[tiab] OR rectal[tiab] OR rectum[tiab] OR bowel[tiab] OR intestin*[tiab]) AND (surgery[tiab] OR surgical[tiab] OR operation[tiab] OR resection[tiab] OR operative[tiab]))

#2

"Prehabilitation"[Mesh] OR prehabilitat*[tiab] OR pre-habilitat*[tiab] OR "pre habilitat*"[tiab] OR "preoperative rehabilitation"[tiab] OR "pre-operative rehabilitation"[tiab] OR "preoperative exercise"[tiab] OR "pre-operative exercise"[tiab] OR "preoperative training"[tiab] OR "pre-operative training"[tiab] OR "preoperative conditioning"[tiab] OR "pre surgery preparation"[tiab] OR "presurgical preparation"[tiab] OR "pre-surgical preparation"[tiab] OR "presurgical intervention"[tiab] OR "pre-surgical intervention"[tiab] OR "preoperative intervention"[tiab] OR "pre-operative intervention"[tiab] OR "preoperative program"[tiab] OR "pre-operative program"[tiab] OR "preoperative preparation"[tiab]

#3

("Multimodal"[tiab] OR "Multi-modal"[tiab] OR "Multi modal"[tiab] OR "Trimodal"[tiab] OR "Three-component"[tiab] OR "Multiple component"[tiab] OR "Combined"[tiab] OR "Comprehensive"[tiab] OR "Integrative"[tiab]) AND (#2)

#4

("Exercise"[Mesh] OR "Exercise Therapy"[Mesh] OR "Physical Fitness"[Mesh] OR "Exercise"[tiab] OR "Physical Activity"[tiab] OR "Training"[tiab] OR "Fitness"[tiab] OR "Physical Therapy"[tiab] OR "Physiotherapy"[tiab] OR "Aerobic"[tiab] OR "Resistance Training"[tiab] OR "Strength Training"[tiab] OR "Endurance Training"[tiab] OR "Walking"[tiab] OR "Physical Conditioning"[tiab] OR "Exercise Prescription"[tiab] OR "Exercise Program"[tiab]) AND (#2)

#5

("Nutrition Therapy"[Mesh] OR "Diet Therapy"[Mesh] OR "Nutritional Support"[Mesh] OR "Dietary Supplements"[Mesh] OR "Protein Supplements"[tiab] OR "Nutrition"[tiab] OR "Nutritional"[tiab] OR "Nutritional Support"[tiab] OR "Nutritional Supplementation"[tiab] OR "Dietary Supplement"[tiab] OR "Diet"[tiab] OR "Dietary"[tiab] OR "Protein"[tiab] OR "Proteins"[tiab] OR "Oral Nutritional Supplement"[tiab] OR "ONS"[tiab] OR "Food Supplement"[tiab] OR "Dietary Counseling"[tiab] OR "Nutritional Counseling"[tiab] OR "Diet Therapy"[tiab] OR "Alimentary"[tiab] OR "Feeding"[tiab] OR "Energy Intake"[tiab] OR "Protein Intake"[tiab] OR "Caloric Intake"[tiab] OR "Malnutrition"[tiab] OR "Nutrition Assessment"[tiab] OR "Enteral Nutrition"[tiab]) AND (#2)

#6

#3 OR #4 OR #5 OR #2

#7

"Randomized Controlled Trial"[pt] OR "Controlled Clinical Trial"[pt] OR "Randomized"[tiab] OR "Randomised"[tiab] OR "Randomly"[tiab] OR "Trial"[tiab] OR "Groups"[tiab] OR "Clinical Trials as Topic"[Mesh] OR "Random Allocation"[Mesh] OR "Therapeutic Use"[sh]

#8

#1 AND #6 AND #7
